# Supplementary figures and images for: Compliance with Covid-19 measures: Evidence from New Zealand
Source: PLoS One. 2022 Feb 9;17(2):e0263376. doi: 10.1371/journal.pone.0263376 (PMC8827475; doi:10.1371/journal.pone.0263376)

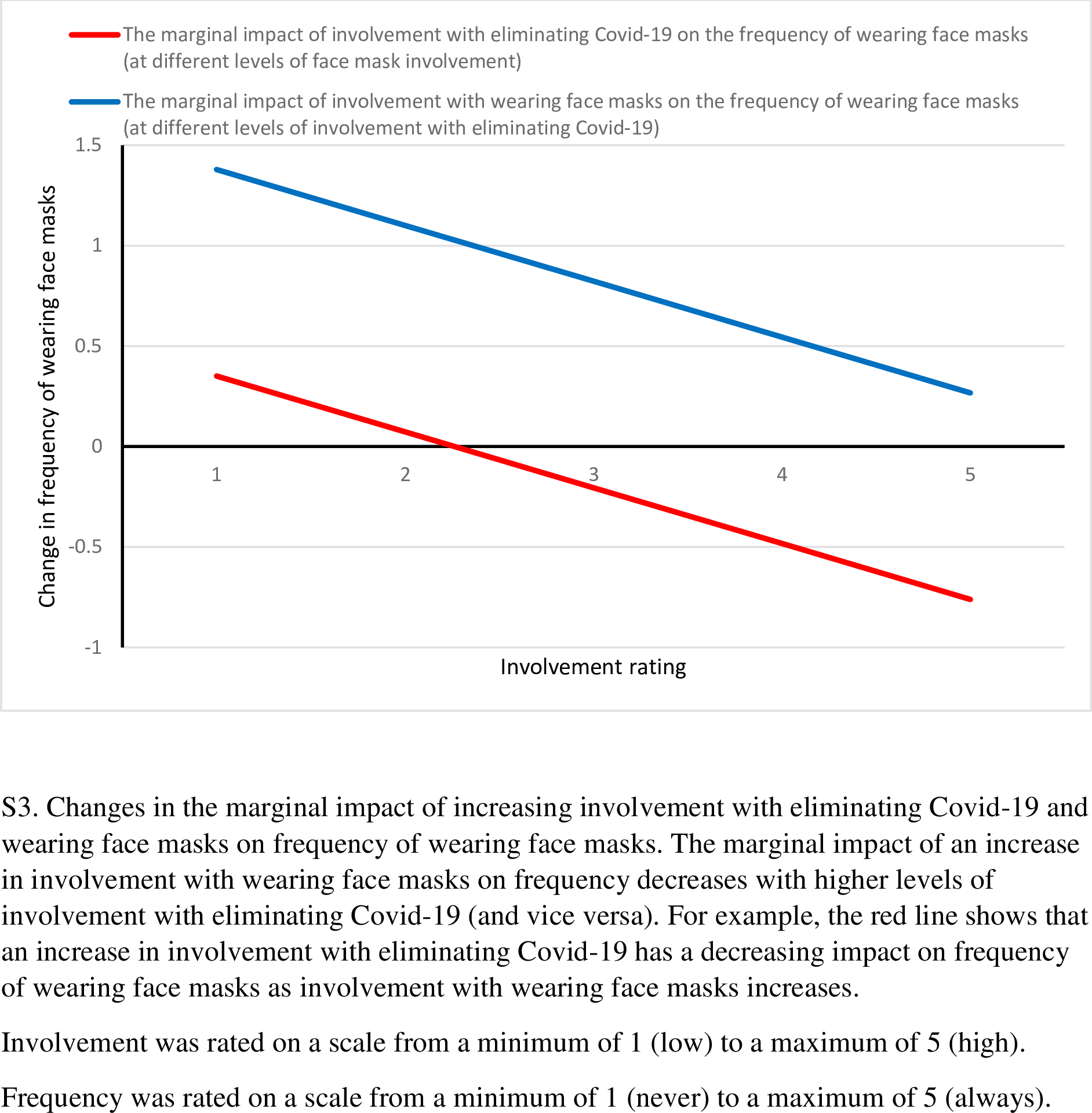

Supplement: S1 Fig — The marginal impact of increase in involvement with wearing face masks on frequency decreases with higher levels of involvement with eliminating Covid-19 (and vice versa). For example, the red line shows that an increase in involvement with eliminating Covid-19 has a decreasing impact on frequency of wearing face masks as involvement with wearing face masks increases. Involvement was rated on a scale from a minimum of 1 (low) to a maximum 5 (high). Frequency was rated on a scale from a minimum of 1 (never) to a maximum 5 (always). (TIF) [file pone.0263376.s001.tif]
