# Supplementary material for: Compliance with Covid-19 measures: Evidence from New Zealand
Source: PLoS One. 2022 Feb 9;17(2):e0263376. doi: 10.1371/journal.pone.0263376 (PMC8827475; doi:10.1371/journal.pone.0263376)
Supplement: S3 File — (PDF) [file pone.0263376.s004.pdf]

Appendix. Summary of sample demographics

Table A1. Age distribution of respondents

Age category	Proportion of respondents	Proportion of Auckland residents[1]
18-29 years	22.8	22.4
30-39 years	21.8	20.3
40-49 years	18.4	18.0
50-59 years	13.1	16.7
60-69 years	12.5	11.9
70 years and over	11.4	10.8

Notes: [1] Source [1]

Table A2. Education distribution of respondents

Education category	Proportion of respondents	Proportion of Auckland residents[1]
Some or all of secondary school	14.2	23.6
Certificate (1-6)	12.4	35.8
Diploma (5-7)	14.3	9.6
Graduate or post-graduate	59.0	31.1

Notes: [1] Source [2]

Table A3. Ethnicity distribution of respondents

Ethnic category	Proportion of respondents	Proportion of Auckland residents[1]
European	53.3	47.5
Māori	4.4	10.3
Pacific Islander	4.7	14.1
Other	37.6	28.1

Notes: [1] Source [52]

Table A4. Income distribution of respondents

Income category	Proportion of respondents	Proportion of Auckland residents[1]
Less than $20,000	4.3	8.1
$20,000 to $50,000	21.2	18.7
$50,000 to $70,000	18.6	11.3
$70,000 to 4100,000	22.0	14.7
More than $100,000	33.8	47.0

Notes: [1] Source [3]

References

1. Stats NZ. Estimated resident population (ERP), subnational population by ethnic group, age, and sex, at 30 June 1996, 2001, 2006, 2013, and 2018 (2020). http://nzdotstat.stats.govt.nz/WBOS/Index.aspx?DataSetCode=TABLECODE7512#

2. Stats NZ. Highest qualification and ethnic group (grouped total responses) by age group and sex, for the census usually resident population count aged 15 years and over, 2006, 2013, and 2018 Censuses (2020). http://nzdotstat.stats.govt.nz/wbos/Index.aspx?_ga=2.69061078.636843804.160211775376 1746062.1551927941#

3. Stats NZ. Household income by region, household type, and source of household income (2020). http://nzdotstat.stats.govt.nz/wbos/Index.aspx?_ga=2.69061078.636843804.1602117753-761746062.1551927941#
